# Today’s data for tomorrow’s knowledge

**DOI:** 10.1093/jamiaopen/ooz010

**Published:** 2019-03-26

**Authors:** Indra Neil Sarkar

**Affiliations:** Center for Biomedical Informatics, Brown University, Box G-R, Providence, Rhode Island 02912, USA

Biomedical Informatics is a transdisciplinary discipline that spans the full range of biomedical applications. There are foundational methodologies that transcend these application areas, which are the basis for functional subgroups of biomedical informatics. Examples of these subgroups include Translational Bioinformatics, Clinical Research Informatics, Clinical Informatics, Patient Informatics, and Public Health Informatics. Linking each of these application areas is the common theme of transforming biomedical or health data into actionable knowledge—that is, the definition of biomedical informatics.

Many an argument has been had, and will likely be had, over what biomedical informatics is (or is not). However, where all who practice biomedical informatics agree is that it is fundamentally about data. Whether they are transformed through computer science, information science, mathematical approaches or assessed quantitatively or qualitatively, all biomedical informaticians agree that data form the basis of knowledge that impacts health and health care. The transformation process of data into knowledge therefore forms the core of any definition of biomedical informatics. It also forms the basis of our tangible contributions to society as biomedical informatics.

Being the Editor of a biomedical informatics journal provides a unique vantage point to all the ways that our community generate, interpret, and use data to improve health and health care. For *JAMIA Open*, the diversity of our content reflects the diversity of our discipline. As you read this issue, you will find various ways that data are contextualized to support the addressing of a biomedical or health challenge. I challenge you to explore new areas that use data that may not be completely familiar. In so doing, you may see new linkages to areas that are indeed familiar and thus expand the horizons for how data may be used.

Alongside the written publication, data represent another aspect of the research that may offer additional insights. To encourage new uses for data, *JAMIA Open* encourages the deposition of data into publicly accessible repositories. I am pleased to note that publications that involve data are associated with datasets that are available through our partner service, the Dryad Digital Repository. The associated publications are then but a chapter in a story for how their associated data may be used or interpreted. Future chapters will be told by others in future studies that reuse or enhance the original data in ways that further advance biomedical insight and impact on health or health care. I invite you to explore the datasets associated with *JAMIA Open*: https://datadryad.org/journal/2574-2531

I sincerely hope that you explore the available datasets and find ways to include them in your future studies. After all, the data would suggest that we have more to gain by sharing.

